# DNA based nanoscale optoelectronic devices enabled by THz driven piezo vibrotronic effect

**DOI:** 10.1038/s41598-025-14152-3

**Published:** 2025-08-11

**Authors:** Samira Fathizadeh, Fatemeh Nemati

**Affiliations:** Department of Physics, Faculty of Science and Modern Technologies, Urmia University of Technology, Urmia, Iran

**Keywords:** Biophysics, Mathematics and computing, Physics, Applied physics, Biological physics, Statistical physics, thermodynamics and nonlinear dynamics

## Abstract

Investigating the piezo-vibrotronics effect in DNA chains, focusing on the interplay between mechanical strain, electronic properties, and photonic interactions paves the way for innovative applications of DNA in nanoscale electronic and optical devices. By applying varying degrees of mechanical deformation to DNA molecules, we observe significant changes in their charge transport properties. Strain-induced polarization within the DNA affects on carrier generation and transport, leading to improved optoelectronic performance. Additionally, photonic excitation under strain conditions demonstrates modulation of electronic responses, highlighting the potential of DNA-based materials in advanced piezo-vibro(photo)tronics devices. I-V characterization and multifractal analysis employ to elucidate these effects, providing a comprehensive understanding of the piezo-phototronics phenomenon in biologic systems.

## Introduction

In the last three decades, the development and production of nanomaterials, along with the exploration of associated technological areas, have progressed rapidly. This phenomenon has elevated nanomaterials to a subject of considerable interest within the global scientific and technological communities. They present many prospective applications across diverse fields such as communications, environmental sciences, energy, material science, chemistry, microelectronics, biology, medical sciences, and various other disciplines^1–3^. In this regard, grounded in the study of piezoelectric semiconductor nanomaterials, Z. L. Wang introduced a novel research domain in 2010, known as piezo-phototronics. This interdisciplinary field combines the principles of piezoelectricity, the intrinsic properties of semiconductors, and the mechanisms of photoexcitation^4,5^. In self-sustaining photodetectors, mechanical stress and strain can influence the processes of charge carrier generation, separation, extraction, and recombination^4,6^. The piezoelectric effect in materials induces an electrical potential when mechanical stress is applied. When this effect is coupled with the photovoltaic effect triggered by incident photons, the resulting phenomenon is termed the piezo-phototronic effect^4^. This effect is beneficial for flexible, wearable self-powered devices, as it has improved performance by up to $\%70$^7^. Therefore, the investigation of the interaction between piezoelectric and photoexcitation properties has led to the development of piezo-photonics^8^. Piezoelectric optoelectronics has laid the basis for the developing novel piezo-phototronic devices^9,10^. Applying piezo-phototronics to various heterojunction devices has created new opportunities for enhancing device performance^11^. Recent studies have shown that the piezo-phototronic effect plays a significant role in solar cells, light-emitting diodes (LEDs), photocatalysis, and, most prominently, photodetectors^12–16^. Piezo-phototronics has been shown to greatly improve the effectiveness of optical devices by adjusting the electronic band structure at the junction by strain fluctuations, this feat is challenging to accomplish with conventional photodetector technologies^17^.

Using the piezotronic and piezo-phototronic effects, innovative sonosensitizers based on piezoelectric semiconductor nanomaterials have shown promising applications in sonodynamic therapy (SDT). On the other hand, developing next-generation tactile sensor matrices or electronic skins is crucial for human-machine interfaces and multifunctional robotics, which can be achieved using the piezo-phototronic technology^18^. These devices must meet critical requirements, including sensitivity, rapid response, and high spatial resolution. Traditional designs of tactile sensor matrices are inadequate for real-time dynamic pressure and strain imaging. The luminescence of LEDs based on piezoelectric semiconductors can be controlled through the piezo-phototronic effect, which means light intensity reacts to applied strain. This principle was employed to create a light-based pressure sensor with a nanowire LED array. Additionally, electroluminescence is well-suited for real-time measurement and high-resolution pressure mapping. Additionally, the piezo-phototronic effect is utilized in electrochemical processes. The efficiency of the photoelectrochemical water splitting process can be greatly improved by adjusting the barrier height at the semiconductor junction interface using strain-induced piezopolarization^19^.

Consequently, piezotronics and piezo-phototronics represent innovative and promising approaches for designing new electronic and optoelectronic devices^20^. While piezotronics and piezophototronics have garnered significant attention as effective methods for enhancing electronic and photoelectric devices, we anticipate that their applications will be further broadened by integrating them with other research fields, such as sensor networks, life sciences, human-machine interface integration, and energy science. In this study, we examine a biomolecular chain derived from DNA to investigate its piezo-vibro(photo)tronic properties. The charge transport properties of DNA chains enable their use as fundamental components in nanoelectronic devices. The significance of electronic transport through nanodevices for nanotechnology motivates the study of charge transport properties of DNA from a physical perspective. Analyzing the piezo-phototronics properties of DNA can also enhance our understanding of its conductivity. Furthermore, comprehending the interaction between the electrical, mechanical, and optical properties of DNA is crucial for the design of molecular nanodevices. Bruot et al. were the first to experimentally demonstrate piezoelectric effect in single DNA molecules^21^. Then, the piezoelectric properties of DNA were theoretically validated^22^. Consequently, DNA has been established as a semi-conducting material exhibiting piezoelectric properties. In this context, we have irradiated a strained DNA chain to form the third edge of the semiconductor, piezoelectricity, and photoexcitation triangle, thereby investigating its piezophototronic properties. Here, we propose the term piezo-vibrotronic effect to describe the coupling between mechanical strain and THz-frequency electromagnetic (EM) waves in biomolecules, distinguishing it from classical piezo-phototronics, which traditionally refers to interactions at optical frequencies. This effect modulates the electrical transport properties by jointly influencing vibrational dynamics (mechanical degrees of freedom) and electron hopping amplitudes through the Peierls phase in the tight-binding Hamiltonian. The piezo-phototronic effect, as established in prior literature (e.g., Wang et al., Nano Today, 2010)^4^, refers to the modulation of optoelectronic processes in piezoelectric semiconductors (e.g., ZnO, GaN) via photoexcitation (optical/UV) and strain-induced polarization. It typically operates at frequencies above 100 THz (visible/UV) and relies on bandgap transitions. In contrast, the Piezo Vibrotronic Effect involves low-frequency THz irradiation ($\sim <2~$THz), which excites vibrational modes in the DNA (twisting, stretching), not interband electronic transitions. A coherent coupling between THz-induced vibrational excitation and mechanical strain jointly modulates hopping integrals and on-site energies, thereby enhancing or suppressing charge delocalization. This defines a regime in which mechanical strain remains dynamically coupled to the THz field-unlike in optical-frequency phototronics, where ultrafast carrier relaxation effectively decouples mechanical effects. Accordingly, the term vibrotronic emphasizes the role of vibrational modes (e.g., collective oscillations in DNA or lattice vibrations) in mediating the strain–THz coupling, analogous to how phototronic denotes light–matter interaction.

Piezo-phototronic devices represent a transformative platform for next-generation biosensors, offering unprecedented sensitivity for disease diagnosis^6^. Piezo-phototronic devices hold significant potential for biological detection and disease diagnostics, particularly when integrated into DNA-based biosensors^23^. The proposed model of EM wave–radiation and strain-modulated charge transport in DNA chains holds significant promise for the development of advanced biological detectors. DNA-based piezo-vibro(photo)tronic sensors could play a pivotal role in disease diagnosis by enabling multi-parameter sensing, enhanced performance optimization, and strong clinical relevance. Such biosensors can simultaneously detect mechanical (strain), optical (photon flux), and electrochemical (charge transport) signals through DNA’s piezo-phototronic response. Strain-gated DNA photodetectors may enable the identification of disease biomarkers by combining optical absorption and mechanical vibration analysis. Current–strain–photon coupling offers a design framework for tuning sensor sensitivity by adjusting the irradiation wavelength to match DNA’s electronic transitions and optimizing strain fields to amplify piezoelectric polarization at critical base-pair sites. Piezo-phototronic detectors can enable early-stage cancer detection through the electromechanical signatures of mutated DNA strands and facilitate pathogen identification via optically enhanced piezoresponse in hybrid DNA–antibody complexes. Unlike conventional fluorescence-based methods, piezo-phototronic biosensors detect biomolecular interactions without the need for external markers. Strain-induced modulation of charge transport further enhances signal detection, making this approach well-suited for low-concentration biomarker analysis. We employ a tight-binding approach to examine the charge transport properties of DNA affected by external mechanical strain and EM wave irradiation. To explore the piezo-vibrotronic effect, we account for the influence of external strain and photoirradiation by incorporating corrections into the tight-binding model. We have considered the electrical response of DNA chains to the strain and THz-wave irradiation through the electrical current flowing through it and I-V characteristic diagrams. Multifractal analysis as a chaos theory tool can help to analyze and confirm the results.

## Model

The EM wave-induced charge transport in a DNA chain consisting of *N* base pairs, sandwiched between left and right leads, is described

1 $$\begin{aligned} \mathscr {H}=\mathscr {H}_{DNA}+\mathscr {H}_{lead}+\mathscr {H}_{DNA-lead}+\mathscr {H}_{photon} \end{aligned}$$

where $\mathscr {H}_{DNA}$ represents the semiclassical Hamiltonian of the DNA molecular chain, which includes the following components:

2 $$\begin{aligned} \mathscr {H}_{DNA}=H_{PBD}+H_{e} \end{aligned}$$

$H_{PBD}$ characterizes the elongation of base pairs along the hydrogen bonds of complementary bases, as described by the Peyrard-Bishop-Dauxois model, as follows^24^:

3 $$\begin{aligned} H_{PBD}=\sum _{n}(\frac{1}{2}m\dot{y}_{n}^{2}+V(y_{n})+W(y_{n},y_{n+1})) \end{aligned}$$

where, $m=300~amu$ is base pair mass, and $y_{n}$ denotes the transverse displacement of base pairs from the equilibrium position. $V(y_{n})=D_{n}(e^{a_{n}y_{n}}-1)^{2}$ represents the Morse potential, which describes the stretching of hydrogen bonds. Meanwhile, $W(y_{n},y_{n+1})=\frac{k}{2}(1+\rho e^{-b(y_{n+1}+y_{n})})(y_{n+1}-y_{n})^2$ denotes the stacking potential along the DNA strand. The parameters of the model and their constant values are detailed in Table 1^25,26^.

**Table 1:** Parameters value and their description in the PBD model.

Parameter	Description	Value
$D_{AT}$	depth of the Morse potential for AT base pairs	0.05 *eV*
$D_{GC}$	depth of the Morse potential for AT base pairs	0.075 *eV*
$a_{AT}$	1/*width* of the Morse potential for AT base pairs	$4.2~A^{-1}$
$a_{GC}$	1/*width* of the Morse potential for GC base pairs	$6.9~A^{-1}$
*k*	stacking constant	$0.04~eV/A^{2}$
$\rho$	intensity of anharmonic interactions	0.5
*b*	damping coefficient	$0.35~A^{-1}$

$H_{e}$ denotes a theoretical analysis based on the quantum mechanical model of charge transport in DNA nanowires, incorporating the effects of piezoelectric potential and photonic excitation written as follows^27,28^:

4 $$\begin{aligned} H_{e}=\sum _{n}\sum _{j}(\epsilon _{n,j}c_{n,j}^{\dagger }c_{n,j} -t_{n,n+1}c_{n+1,j}^{\dagger }c_{n,j}+H.c.)-\sum _{n}(t_{j,\acute{j}}e^{-py_{n}}c_{n,1}^{\dagger }c_{n,2}+H.c.) \end{aligned}$$

where, $c_{n,j}^{\dag }$ and $c_{n,j}$ are the creation and annihilation operators of an electron in site *n* of strand *j* of DNA. $\epsilon _{n,j}$ is the on-site energy of an electron in site (*n*, *j*) chosen as $\epsilon _{A}=-0.07$, $\epsilon _{T}=0.83$, $\epsilon _{C}=0.56$, and $\epsilon _{G}=-0.56$ in *eV*. $t_{n,n+1}=0.2$ and $t_{j,\acute{j}}=0.15$ in *eV* are the intrastrand and interstrand hopping constants, respectively^28^. The exponential function, with a cutoff constant of ($p = 0.2~\text {{\text{\AA }}}^{-1}$), defines the exponential coupling between charge and lattice in the interstrand term.

The electrical potential difference $V_{sd}$ can be applied to the DNA strand via the left and right connections. $\mathscr {H}_{lead}+\mathscr {H}_{DNA-lead}$ illustrate the Hamiltonian of leads and their interaction with DNA, respectively, as follows^29,30^:

5 $$\begin{aligned} {\mathcal{H}}_{{lead}} + {\mathcal{H}}_{{DNA - lead}} = & \sum\limits_{K} {\sum\limits_{J} {[(\epsilon _{{L_{{k,j}} }} + \frac{{eV_{{sd}} }}{2})a_{{L_{{k,j}} }}^{\dag } a_{{L_{{k,j}} }} } } \\ + & \,(\epsilon _{{R_{{k,j}} }} - \frac{{eV_{{sd}} }}{2})a_{{R_{{k,j}} }}^{\dag } a_{{R_{{k,j}} }} ] + \sum\limits_{k} {\sum\limits_{j} {(t_{L} a_{{L_{{k,j}} }}^{\dag } c_{{1,j}} + t_{R} a_{{R_{{k,j}} }}^{\dag } c_{{N,j}} + H.c.)} } \\ \end{aligned}$$

where $a_{L(R)_{k,j}}^{\dagger }(a_{L(R)_{k,j}})$ is the creation (annihilation) operator of an electron in site (*k*, *j*) of lead *L*(*R*). $\epsilon _{L(R)_{k,j}}=7.75~eV$ and $t_{L(R)}=0.42~eV$ are the on-site energy of leads and the hopping energy between the leads and DNA, respectively^29^.

The simulation of THz irradiation on a DNA chain can be achieved by incorporating a Peierls phase into the intrastrand hopping term. Consequently, the interaction between electrons and photons is expressed as follows^28,31^:

6 $$\begin{aligned} \mathscr {H}_{photon}=\sum _{n}\sum _{j}[-t_{n,n+1}\exp (\frac{ier_{0}}{\hbar c}A(t))c_{n+1,j}^{\dagger }c_{n,j}+H.c.] \end{aligned}$$

where, $A(t)=A_{0}\cos (\omega t) \exp (-\frac{(t-t_{c})^{2}}{2\tau ^{2}})$ is the potential vector. In this context, $A_0$, $\omega$, $t_c$, and $\tau$ represent the amplitude, the oscillation frequency, the pulse center, and the pulse half-width, respectively.

In the presence of mechanical tension, we have considered stretching as one of the DNA’s responses to external strain. Unlike conventional bulk materials, DNA is regarded as a macroscopic, uniform, and isotropic flexible rod with a circular cross-section that undergoes transverse expansion when stretched^32^. Following the theory of linear elasticity, we have elucidated the relationship between transverse strain ($S_{t}$) and applied longitudinal strain ($S_{l}$) by employing a negative Poisson’s ratio ($\sigma = -0.5$). This relationship is mathematically expressed as $S_{t} = -\sigma S_{l}$^33,34^. When DNA is positioned between two contacts and is not aligned with the electric field between them, results in the emergence of a perpendicular field component relative to the molecule’s axis. This perpendicular field generates a gating effect, which can be described by the equation $V_g = 2E_{\bot }r$, where $E_{\bot }$ denotes the orthogonal electric field and 2*r* represents the equivalent strand diameter^35^. For this oriented DNA configuration, the gate voltage ($V_{g}$) depends on the source-drain voltage ($V_{sd}$) and is given by the equation $V_{g} = \frac{2r}{L} \tan (\alpha ) V_{sd}$, where *L* represents the DNA length and $\alpha$ (ranging from 0 to $\frac{\pi }{2}$) denotes the tilt angle of the DNA. Within a DNA strand subjected to a orthogonal electrical field, the on-site energy at each site undergoes modification as^35,36^:

7 $$\begin{aligned} \tilde{\epsilon }_{n,j}=\epsilon _{n,j}+\frac{1}{2}eV_{g}\cos (\frac{2n\pi }{10}+\phi _{0}) \end{aligned}$$

where $\epsilon _{n,j}$ denotes the on-site energy of the *n*th site in the absence of an electric field, and *e* denotes the electron charge. The helical structure of DNA strands under strain induces the subsequent modification to the on-site energies:

8 $$\begin{aligned} \tilde{\epsilon }_{n,j}=\epsilon _{n,j}+\frac{e\tilde{r}}{\tilde{L}}\tan (\alpha ) V_{sd}\cos (\frac{2n\pi }{10}+\phi _{0}) \end{aligned}$$

where $\tilde{r} = r \left( 1 - \sigma \frac{S_{l}}{100}\right)$ and $\tilde{L} = L \left( 1 + \frac{S_{l}}{100}\right)$ represent the optimized radius and length of the DNA under strain, respectively^36^. $\phi _{0}$ represents the phase difference between the electric field and the first base pair.

Conversely, the strain-dependent hopping constants are determined using the following correction^36^:

9 $$\begin{aligned} \tilde{t}_{n,n+1}=-t_{n,n+1}\exp (-(1+\frac{S_{l}}{100})) \end{aligned}$$

**Fig. 1:**
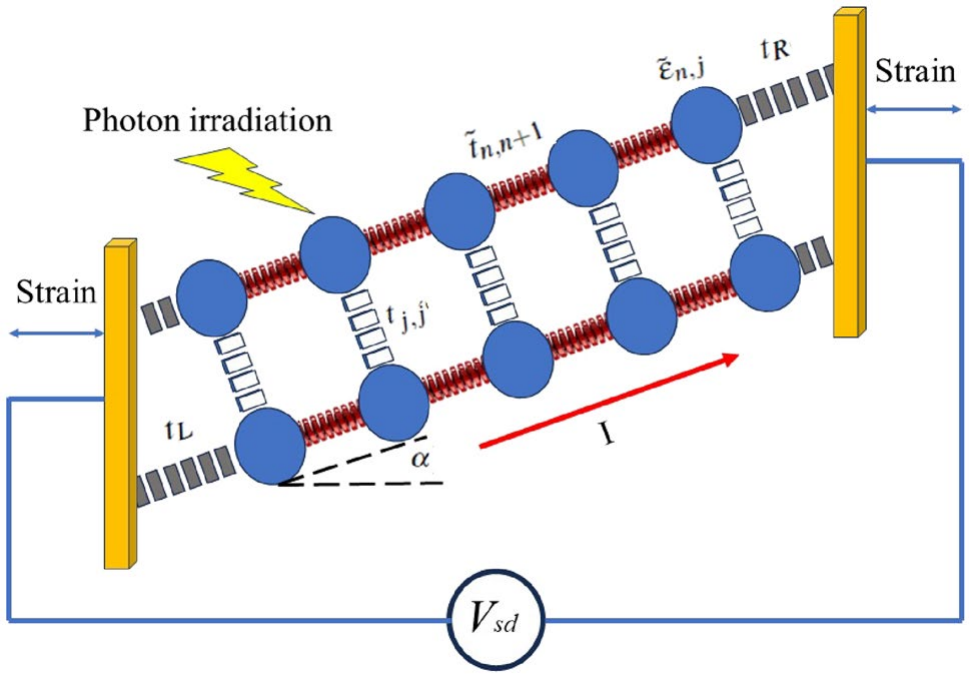
Schematic representation of charge transport model of a DNA chain connected to the leads with electrical potential difference of $V_{sd}$. blue circles show DNA bases irradiated by the THz EM wave. DNA chain can be compressed and stretched via the external strain. The on-site energies and hopping parameters are presented.

In the present relations, $S_{l} < 0$ indicates compression strain, while $S_{l} > 0$ signifies stretching strain. A schematic illustration of electronic part of model is provided in the Fig. 1.

To explore the dynamical behavior of the system, Hamilton’s equations $(\dot{p}_{n}=-\frac{\partial \mathscr {H}}{\partial \dot{q}_{n}})$ are employed to identify fluctuations in the base pairs, where $p_{n}$ represents the momentum and $q_{n}$ denotes the coordinates. Concurrently, the Heisenberg approach $(\dot{c}_{n}=-\frac{i}{\hbar }[c_{n},\mathscr {H}])$ is utilized to determine the charge dynamics. The evolution equations obtained from the Hamiltonian system are as follows:

10 $$\begin{aligned} m\ddot{y}_{n}= & 2a_{n}D_{n}e^{-a_{n}y_{n}}(e^{-a_{n}y_{n}}-1)\nonumber \\+ & \frac{kb\rho }{2}[e^{-b(y_{n}+y_{n-1})}(y_{n}-y_{n-1})^{2}+e^{-b(y_{n+1}+y_{n})}(y_{n+1}-y_{n})^{2}]\nonumber \\- & k[(1+\rho e^{-b(y_{n}+y_{n-1})}(y_{n}-y_{n-1})-(1+\rho e^{-b(y_{n+1}+y_{n})}(y_{n+1}-y_{n})]\nonumber \\- & 2p t_{j,\acute{j}}\exp (-py_{n})|<c_{n,1}^{\dagger }c_{n,2}>| \end{aligned}$$

11 $$\begin{aligned} i\hbar \dot{c}_{n,j}= & \tilde{\epsilon }_{n,j}c_{n,j}-\tilde{t}_{n,n+1}\exp (\frac{ier_{0}}{\hbar c}A(t))(c_{n+1,j}+c_{n-1,j}) -t_{j,\acute{j}}\exp (-py_{n})(c_{n,j}(\delta _{j,1}+\delta _{j,2}))\nonumber \\+ & \sum _{k}(\delta _{n,1}t_{L}a_{L_{k,j}}+\delta _{n,N}t_{R}a_{R_{k,j}}) \end{aligned}$$

12 $$\begin{aligned} i\hbar \dot{a}_{L_{k,j}}=(\epsilon _{L_{k,j}}+\frac{eV_{sd}}{2})a_{L_{k,j}}+t_{L}c_{1,j} \end{aligned}$$

13 $$\begin{aligned} i\hbar \dot{a}_{R_{k,j}}=(\epsilon _{R_{k,j}}-\frac{eV_{sd}}{2})a_{R_{k,j}}+t_{R}c_{N,j} \end{aligned}$$

## Electrical current

Electrical current provides a robust framework for analyzing the transport properties of molecular systems. By incorporating the effects of external fields, intra-molecular interactions, and boundary conditions, we can determine charge transport properties of DNA which has implications for molecular electronics, nano and bioelectronics. Also, investigating the influence of external stimulus such as tension and electromagnetic fields on the electronic properties of DNA, is relevant for applications in sensing and signal processing. Using the continuity equation of electrical charge and current density ($\frac{\partial \rho }{\partial t} + \nabla \cdot \textbf{J} = 0$), we can derive the local electrical current operator in every site at time *t*, as follows:

14 $$\begin{aligned} I_{n,j}(t)= & \frac{ie}{\hbar }[\tilde{t}_{n,n+1}\exp (\frac{ier_{0}A(t)}{\hbar c})(c_{n+1,j}^{\dag } c_{n,j}-c_{n-1,j}^{\dag }c_{n,j})\nonumber \\+ & t_{j,\acute{j}}\exp (-py_{n})(c_{n,2}^{\dag }c_{n,1}-c_{n,1}^{\dag }c_{n,2})\nonumber \\+ & \sum _{k}(t_{L}(c_{n,j}^{\dag }a_{L_{k,j}}-a_{L_{k,j}}^{\dag }c_{n,j})\delta _{n,1} -t_{R}(c_{n,j}^{\dag }a_{R_{k,j}}-a_{R_{k,j}}^{\dag }c_{n,j})\delta _{n,N})] \end{aligned}$$

Then, the total electrical current is defined as $I(t)=\sum _{n,j}I_{n,j}(t)$.

## Results

We have analyzed electrical current flowing through DNA under varying mechanical strains and irradiation conditions to investigate the piezo-vibrotronic effect in DNA nanowires. The aim is to elucidate the underlying factors that govern the interaction between piezoelectric and photonic properties in DNA-based nanostructures.

### Electrical response under mechanical strain (isolated piezoelectric effect)

Investigating of the piezoelectric effect in DNA nanowires involves an analysis of the electrical current behavior under external mechanical strain. In the current study, we have considered a *P*53 sequence of DNA with $N=120~bps$ to determine the effect of the mechanical strain on its charge transport properties.

**Fig. 2:**
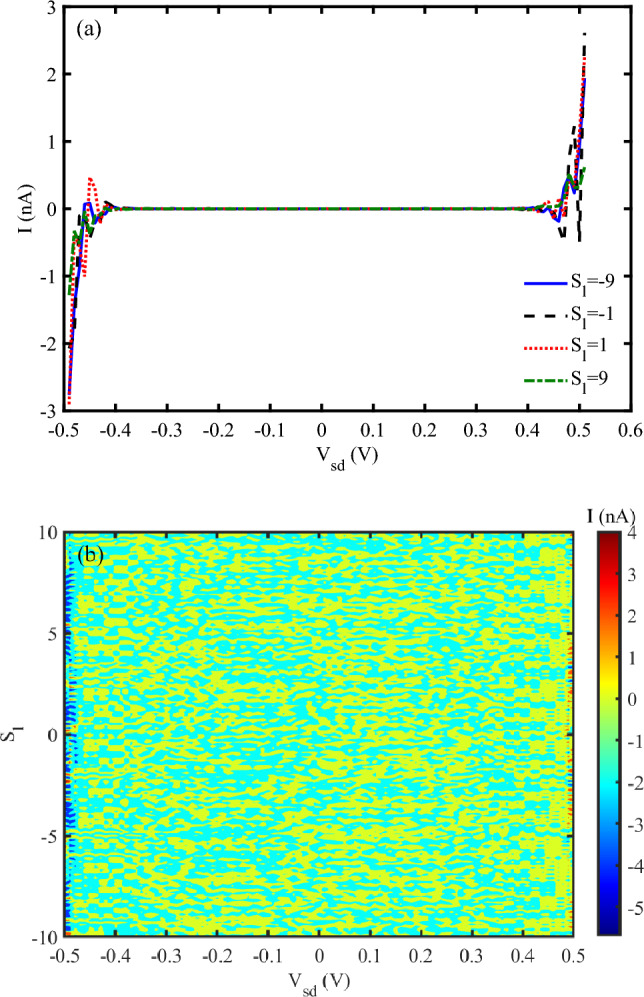
(**a**) I-V characteristic diagram for different strain effects (without THz irradiation), (**b**) Electrical current following through DNA versus simultaneous variation of source-drain voltage and strain (without THz irradiation).

Figure 2 a, shows I-V characteristic diagram for different strain effects in low voltage conditions. At the voltages up to about $V_{sd}=0.4~V$, no electrical current flows through the system at direct and reverse voltages. When, the voltage reaches 0.4 *V* and higher than it, the electrical current in the system oscillates and increases nonlinearly. The electrical response of the DNA sequence to the mechanical strain is different. Conversely, the electrical current behavior of the compressing strain $(S_{l}<0)$ and stretching one $(S_{l}>0)$ is different. Compressive strain $(S_{l}<0)$ shortens the DNA helix, reducing the distance between adjacent nucleobases. This increases electronic overlap between $\pi -$stacked bases, enhancing charge carrier mobility. However, excessive compression may distort the hydrogen bonds or backbone, possibly disrupting charge transport pathways. Tensile strain $(S_{l}>0)$ elongates the DNA, increasing the distance between bases. This reduces $\pi -$orbital overlap, decreasing hopping efficiency and thus lowering current. If the applied strain becomes too large, the DNA may denature, breaking conduction pathways entirely. DNA exhibits piezoelectric behavior due to its asymmetric charge distribution. Under compressive strain, the piezoelectric effect can enhance internal polarization, modify the local electric field, and facilitate charge transport. In contrast, tensile strain may reduce polarization, thereby hindering current flow.

Additionally, strain alters the electronic band structure of DNA. Compression can narrow the bandgap, increasing carrier density and improving conductivity, while tensile strain may widen the bandgap, reducing charge transport efficiency. According to Ref.^37^, the threshold voltage, where significant current begins to flow, shifts with varying strain. Under low strain, the threshold voltage is lower, while higher strain values increase the threshold voltage, reflecting the increased energy barrier for charge transport. At low strain values, DNA retains its native helical structure, resulting in relatively stable and high conductivity. The current–voltage (I–V) curve in this regime exhibits a linear relationship, indicating ohmic behavior. As strain increases, the DNA begins to deform, altering the electronic coupling between base pairs. This leads to a non-linear current–voltage (I–V) characteristic, with a noticeable increase in current at higher voltages. At high strain levels, significant structural distortions disrupt charge transport pathways. The I–V curve in this regime exhibits a reduced slope, indicating decreased conductivity and potential saturation effects^33^. Figure 2 b is a 3-D diagram that shows the electrical current through DNA for simultaneous variation of source-drain voltage and strain values. It confirms that electrical current shows different behavior for different applied strains after $V_{sd}=0.4~V$. For lower voltages, the electrical current vanishes.

The obtained results are in agreement with previous findings that reported that I-V curves of the DNA nanowires exhibited significant changes when subjected to mechanical strain. Under compressive strain, an increase in current was observed, indicating enhanced carrier mobility due to the piezoelectric potential generated within the DNA structure^38^.

### Charge transport under THz radiation (isolated vibronic effect)

**Fig. 3:**
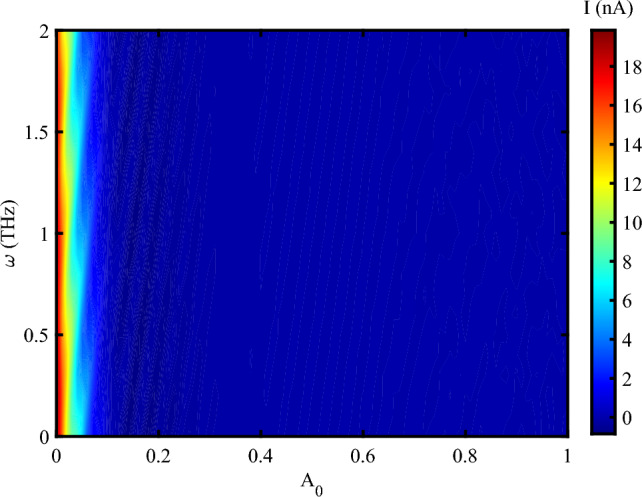
Electrical current following through DNA versus concurrent alteration of both the amplitude of EM irradiation in the unit of $\frac{\hbar c}{er_{0}}$ and its frequency at unit of *THz* without strain effect, ($V_{sd}=0.1~V$).

EM wave irradiation reveals significant changes in the electrical properties of DNA. The previous results indicate a clear correlation between EM wave intensity and the variation in charge transport efficiency. In the present research, we examine the effect of EM wave irradiation on the electrical current through DNA in the absence of an external strain (Fig. 3).

In this work, the electromagnetic (EM) wave excitation spans frequencies below $2~\text {THz}$ (millimeter-wave to far-infrared), targeting DNA’s low-energy vibrational modes rather than electronic transitions. This contrasts with classical piezo-phototronics, which typically operates at UV–visible frequencies-for example, ZnO bandgap excitation occurs at approximately $3.2~\text {eV}$ ($\sim 385~\text {THz}$). We selected THz frequencies in the context of DNA piezo-phototronics because DNA’s collective vibrational modes (e.g., twisting and stretching) resonate within the 0.1–$2~\text {THz}$ range^39,40^. These vibrational modes enable strain–photon coupling without damaging covalent bonds, unlike ultraviolet (UV) irradiation. Terahertz (THz) waves modulate hopping integrals through lattice vibrations, while mechanical strain alters the piezoelectric polarization. In contrast, optical frequencies primarily excite interband electronic transitions, where ultrafast carrier dynamics tend to decouple the effects of mechanical strain. Terahertz (THz) frequencies match DNA’s collective vibrational modes-such as twisting and breathing-typically in the 0.1–$1~\text {THz}$ range, thereby enabling effective strain–photon coupling. Below $2~\text {THz}$, piezoelectric polarization remains coherent and intact. In contrast, optical frequencies ($>10~\text {THz}$) can disrupt covalent bonds, whereas THz radiation selectively excites delocalized charges without causing structural damage. Ultraviolet (UV) photons (4–$6~\text {eV}$) excite localized $\sigma$ and $\pi$ electrons, disrupting resonant charge transport pathways. Moreover, mechanical strain cannot respond to the ultrafast optical cycles (periods $<10~\text {fs}$), effectively nullifying piezoelectric modulation at such frequencies. High-frequency excitation also promotes non-radiative decay processes, leading to thermal heating that can raise DNA temperatures above its melting point ($T > 350~\text {K}$). As a result, the 0–$2~\text {THz}$ range optimally supports piezo-phototronic (or piezo-vibrotronic) coupling while avoiding bond-breaking energy thresholds. In contrast, at optical frequencies, interband electronic transitions and thermal effects dominate, effectively decoupling strain-mediated charge transport.

Figure 3, shows the electrical current through DNA sequence versus concurrent alteration of both the amplitude and frequency of the irradiating EM wave in the absence of an external mechanical strain. At the low amplitude irradiation, the electrical current oscillates with the frequency and reaches about 18 *nA*. However, the electrical current shows a decreasing behavior concerning the THz amplitude as far as the electrical current vanishes when the amplitude of photon reaches 0.1 $\frac{{\hbar c}}{{er_{0} }}$.

### Combined strain and THz excitation (piezo-vibrotronic effect)

**Fig. 4:**
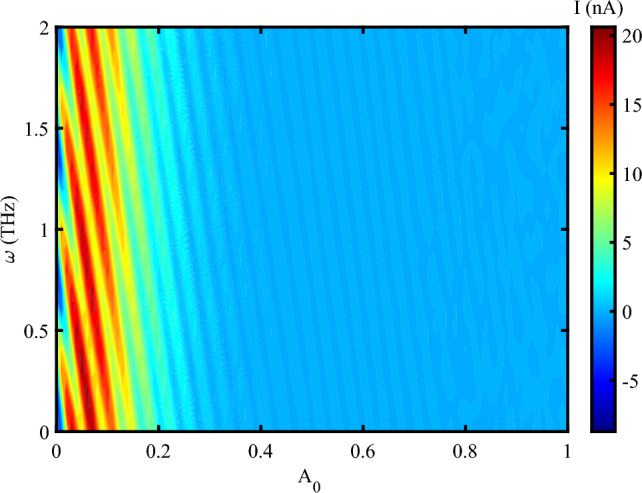
Electrical current following through DNA versus concurrent alteration of both the amplitude of EM irradiation in the unit of $\frac{\hbar c}{er_{0}}$ and its frequency at unit of *THz* at $S_{l}=2$, ($V_{sd}=0.1~V$).

When the system is affected by an external strain in the presence of THz radiation, we have an oscillating electrical current at different frequencies up to $A_{0}=0.3~\frac{\hbar c}{er_{0}}$ (Fig. 4). After this value, the electrical current decreases, but doesn’t vanishes. The result determines that the photon irradiation enhances the electrical current through DNA. This indicates that the electrical feedback of DNA to the irradiation emerges as an amplified electrical current. Also, via the applying a mechanical strain, the electrical current can flow through DNA at the higher amplitudes and reach to 20 *nA* at some frequencies. Mechanical strain modulates the distance and orbital overlap between $\pi$-stacked nucleobases. An optimal level of strain-neither too weak nor too strong-maximizes electronic coupling, thereby enhancing charge delocalization. At a critical strain value, the system reaches a resonant condition in which charge carriers hop more efficiently between bases. As a result, the electrical current reaches its maximum.

**Fig. 5:**
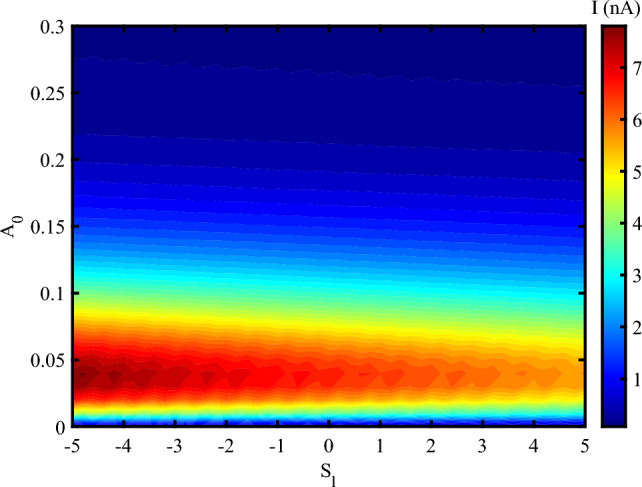
Electrical current following through DNA versus simultaneous variation of EM amplitude in the unit of $\frac{\hbar c}{er_{0}}$ and strain intensity, ($V_{sd}=0.2~V, \omega =0.02~THz$).

In Fig. 4, the current exhibits periodic oscillations with the electromagnetic (EM) frequency $\omega$, but eventually saturates at a maximum value that becomes independent of $\omega$. Electronic states in DNA are excited when the electromagnetic frequency matches the inter-base hopping rates (approximately 0.1–$2~\text {THz}$ for $\pi$-stacked DNA). This resonance leads to the formation of standing charge waves, resulting in current oscillations. However, at higher frequencies, strain-induced polarization saturates due to the finite mechanical response time of DNA (on the order of $\sim 100~\text {fs}$). Therefore, the periodic oscillations in current arise from resonant charge injection into the electronic states of DNA, while the maximum current is determined by the time scales of carrier recombination and piezoelectric screening. Additionally, the observed current oscillations result from the modulation of electron hopping integrals by the interaction between the applied THz field and DNA’s low-frequency vibrational modes. When the driving frequency resonates with specific vibrational modes-typically in the 0.1–$2~\text {THz}$ range-constructive interference enhances hopping coherence, leading to oscillatory behavior in the charge current. Considering the simultaneous effect of photon irradiation and strain, one can report that the charge transport in DNA decreases due to increase the amplitude of irradiation (Fig. 5). Electrical current amplifies when a moderate stretching strain applies on DNA sequence. Electrical current flowing through DNA in the presence of a compressing strain is higher than a stretching strain. The application of tensile strain resulted in a decrease in current, suggesting a modulation of the band structure and carrier transport properties^41^. The piezoelectric potential generated by the applied strain influenced the charge carrier dynamics. This potential modulated the energy barriers at the lead-DNA interfaces, thereby affecting the injection and extraction of carriers^42^. According to the previous reports, the strain-induced piezoelectric potential also altered the local electric field within the DNA nanowires, impacting the recombination and separation of photo-generated electron-hole pairs^43^. Under illumination, the DNA nanowires demonstrated a notable increase in photocurrent when mechanical strain was applied. This enhancement is attributed to the piezo-phototronic effect, where the piezoelectric potential aids in the separation and transport of photo-generated carriers^44^. The strain-dependent photocurrent response highlights the synergistic interaction between the piezoelectric and photonic properties of the DNA nanowires^45^. The analysis revealed that the piezo-vibro(photo)tronic effect led to a modulation of the band structure in the DNA nanowires. The strain-induced piezoelectric potential caused a shift in the conduction and valence bands, thereby influencing the carrier transport mechanism^46^. This band structure modulation is crucial for optimizing the performance of DNA-based optoelectronic devices, as it allows for precise control over the electronic properties through mechanical strain^47^.

**Fig. 6:**
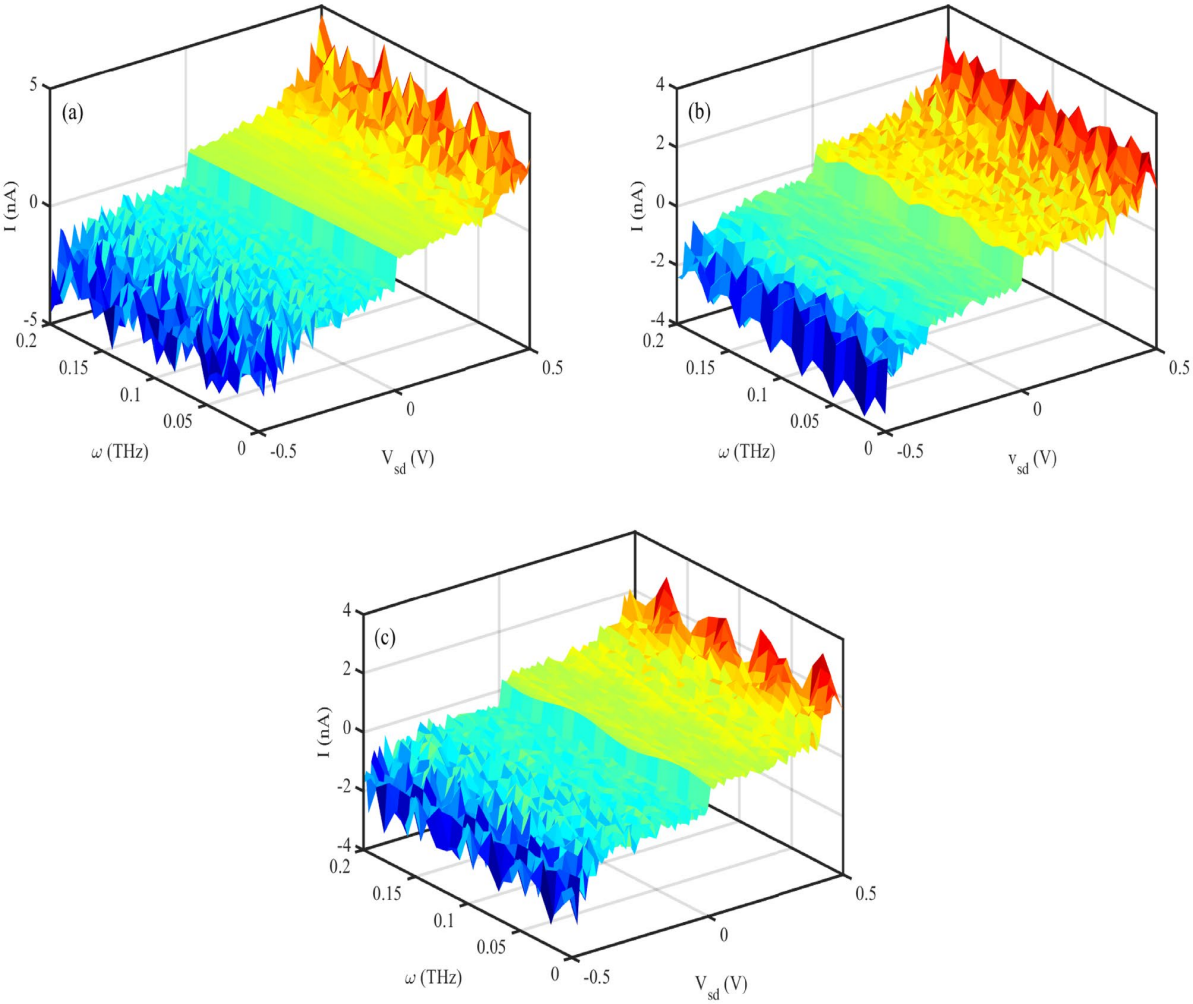
Electrical current versus simultaneous variation of photon frequency and source-drain voltage for P53 sequences with (**a**) $N=60~bps$ (**b**) $N=120~bps$ (**c**) $N=180~bps$, ($\omega =0.2~THz, A_{0}=0.8~\frac{\hbar c}{er_{0}}, S_{l}=2$). The images are generated using MATLAB R2016a (https://www.mathworks.com/products/matlab.html).

### Sequence dependence and tilt angle

The effectiveness of charge movement through the DNA molecule can vary with the length of the DNA sequence. The resistance of DNA increases exponentially with the length of the sequence, then, the electrical current decreases due to increasing the DNA length. We have considered the effect of DNA length on I-V characteristic diagrams of the P53 sequence with different lengths (Fig. 6). Fig. 6 a–c shows electrical current flowing through the P53 sequence with (a) $N=60~bps$ (b) $N=120~bps$, and $N=180~bps$ versus simultaneous variation of EM frequency and source-drain voltage. The obtained result determines that the electrical current shows an oscillatory behavior concerning the photon frequency, and decreases via the increasing the sequence length.

**Fig. 7:**
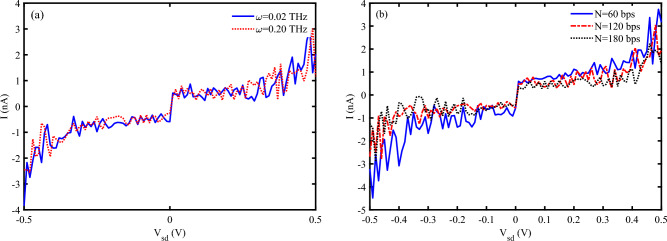
I-V characteristic diagram for (**a**) P53 sequence with $N=120~bps$ at two different frequencies of EM irradiation, (**b**) P53 sequence with different lengths ($\omega =0.2~THz$), ($A_{0}=0.8~\frac{\hbar c}{er_{0}}, S_{l}=2$).

The non-linear I-V characteristics observed at higher strain levels suggest the presence of complex charge transport mechanisms, possibly involving tunneling and hopping processes. These mechanisms are influenced by the altered electronic band structure and the increased disorder within the DNA molecule^37^. Comparing the I-V characteristics of DNA under different strain values helps understand the impact of mechanical deformation on its electronic properties. This analysis can reveal how strain affects the conductivity, threshold voltage, and overall charge transport mechanisms^48^. I-V characteristic diagram of P53 sequence with $N=120~bps$ at two different values of photon frequency is shown in Fig. 7 a. It is clear that the pick point of oscillatory electrical current at $\omega =0.2~THz$ is higher than $\omega =0.02~THz$ at most. Therefore, we can report that the charge mobility of DNA increases when the frequency of photon increases. The increase in charge mobility observed in DNA within the 0.02–$0.2~\text {THz}$ frequency range can be attributed to resonant coupling between THz photons and DNA’s low-energy collective vibrational modes. This coupling enhances charge delocalization along the $\pi$-stacked base pairs. Notably, DNA exhibits intrinsic vibrational modes in the low-frequency range of approximately 0.01–$1~\text {THz}$. When the THz photon frequency matches these intrinsic vibrational modes, resonant absorption occurs, exciting coherent vibrational motion within the DNA structure. These vibrations transiently modulate the electronic coupling between $\pi$-stacked bases, thereby enhancing charge mobility. Specifically, photons in the 0.1–$0.2~\text {THz}$ range can drive coherent lattice dynamics, which reduce dynamic disorder. As a result, the transient improvement in $\pi$-orbital overlap facilitates more efficient charge hopping. The I-V characteristic diagram of P53 sequence with different lengths confirms that the electrical current is inversely related to its length, so that, the electrical current peaks for $N=60~bps$ is the highest among them (Fig. 7 b).

**Fig. 8:**
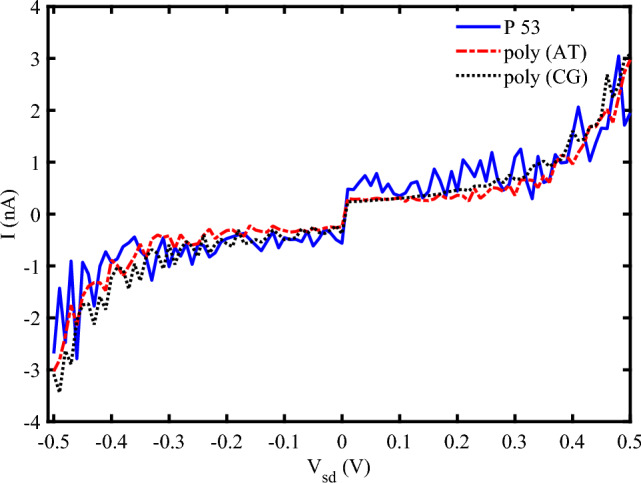
I-V characteristic diagram for Different sequences ($N=120~bp$, $\omega =0.2~THz$, $A_{0}=0.8~\frac{\hbar {c}}{er_{0}}, S_{l}=2$).

The specific order of nucleotides is crucial in charge transport properties of DNA sequences. We have analyzed the influence of several sequences of base pairs with the same length ($N=120~bps$) in the electrical response of DNA to the strain and irradiation in Fig. 8. The general form of the I-V characteristic diagram for three different sequences is almost the same, but, the electrical current peaks are different for them. Poly AT sequences generally exhibit lower charge transport efficiency compared to poly CG sequences due to weaker $\pi -\pi$ stacking interactions between adenine and thymine bases. Also, when subjected to mechanical strain, poly AT sequences tend to elongate more easily, which can disrupt the $\pi -\pi$ stacking interactions and reduce electrical conductivity, but, poly CG sequences are more resistant to mechanical deformation, maintaining better electrical conductivity under strain^49,50^. On the other hand, poly AT sequences can generate photocurrent when irradiated with EM waves, but the efficiency is generally lower compared to poly CG sequences. Poly CG sequences are more efficient at generating photocurrent due to better electronic coupling between bases. These sequences can undergo significant structural changes upon THz irradiation, which can enhance or modulate their electrical properties^51^. The other sequences such as P53 have some regions potentially maintaining conductivity while others may experience significant disruption. Then, the efficiency of photocurrent generation in such a sequence depends on the specific nucleotide arrangement and the presence of photon-sensitive regions. Therefore, this sequence can exhibit varied responses to the strain and photo irradiation, with some regions showing enhanced electrical properties while others may not.

**Fig. 9:**
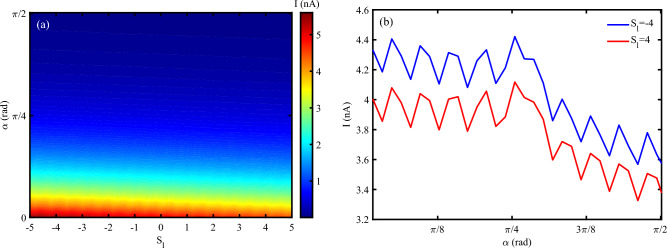
(**a**) Electrical current following through DNA versus simultaneous variation of tilt angle and strain intensity, (**b**) Electrical current versus tilt angle for different strain effects ($N=120~bp, \omega =0.02~THz, A_{0}=0.1~\frac{\hbar c}{er_{0}}, V_{sd}=0.2$).

The tilt angle of the DNA can influence the degree of mechanical deformation. Here, we study the simultaneous effect of variation of tilt angle and strain intensity influences on the electrical current traversing the P53 sequence (Figs. 9 a, b). The electrical current in DNA decreases by increasing the tilt angle. The compressing strain can enhance the electrical current until $\alpha$ is less than $\pi /4~rad$. For $\alpha > \pi /4$, electrical current goes to vanish by increasing the $\alpha$. The result is in agreement with the previous finding. A larger tilt angle may impose additional strain on the hydrogen bonds between base pairs, potentially reducing conductivity^52^. However, a moderate tilt angle can also help maintain the integrity of $\pi$–$\pi$ stacking interactions, thereby preserving electrical conductivity even under mechanical deformation.

### Chaos theory dynamics: multifractal analysis (as an advanced tool)

It was previously reported that the electrical states of a DNA sequences show the multifractal behavior. Also, the electrical current time series flowing through DNA behaves as a multifractal system. Multifractalitiy refers to the property of a time series that exhibits multiple scaling behaviors, meaning different parts of the series may scale differently. This contrasts monofractal series, which have a single scaling exponent. Multifractal analysis is a known method to study the system’s multifractality. In this regard, multifractal spectrum ($D_{q}$), which describes how the fractal dimension of the time series varies with the moment order *q*, is defined as follows:

15 $$\begin{aligned} D_{q}=\frac{1}{q-1}\lim _{\epsilon \rightarrow 0} \frac{\log \sum _{i=1}^{N_\epsilon } \left[ P(i, \epsilon )\right] ^q}{\log \epsilon } \end{aligned}$$

where $\epsilon$ is the scale, $N_\epsilon$ is the number of segments of size $\epsilon$, and $P(i,\epsilon )$ represents the fluctuation function.

On the other hand, the singularity spectrum ($f(\alpha )$) determines the distribution of singularities in the time series. It provides a detailed characterization of the multifractal nature of the series. $f(\alpha )$ is written as follows:

16 $$\begin{aligned} f(\alpha ) = q \alpha - \tau (q) \end{aligned}$$

where $\alpha$ is the singularity strength, and $\tau (q)=(q-1) D_{q}$ is the mass exponent function.

Also, the thermodynamic formulation of multifractal measures can propound an expression for the “analogous” specific heat as follows^22,53^:

17 $$\begin{aligned} C(q)=-\frac{\partial ^{2}\tau (q)}{\partial q^{2}}\approx \tau (q+1)-2\tau (q)+\tau (q+1) \end{aligned}$$

**Fig. 10:**
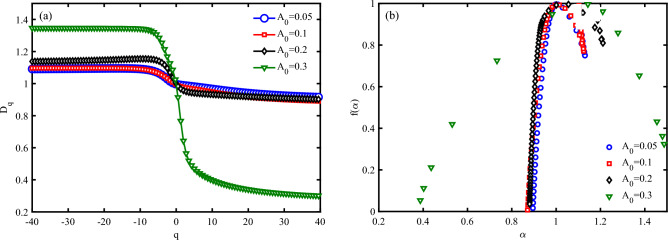
Multifractal analysis of electronic states a P53 sequence with $N=120~bps$ for different irradiation amplitudes in the unit of $\frac{\hbar c}{e r_{0}}$, (**a**) multifractal spectrum (*Dq*), and (**b**) singularity spectrum $(f(\alpha ))$ ($\omega =0.1~THz$, $S_{l}=-4$).

Figures 10 investigate the multifractal spectrum ($D_{q}$) and singularity spectrum ($f(\alpha )$) for different values of EM wave amplitude at a moderate strain effect ($S_{l}=2$) (to check and verify the obtained result of Fig. 5). It is clear that the width of the multifractal spectrum $(D_{q_{max}}-D_{q_{min}})$ increase via the increasing of amplitude of wave (Fig. 10 a). The width of the multifractal spectrum indicates the range of scaling behaviors present in the time series. A larger width suggests a more complex and heterogeneous structure, while a smaller width indicates a more homogeneous structure. From fig. 5, we determine that the electrical current through DNA decreases via the increase of EM wave amplitude and then vanishes. Here, we reported that the decrease in current and its vanishing correspond to increase of width of the multifractal spectrum and complexity of the system. On the other hand, the width of the $f(\alpha )$ function, often denoted as ($\Delta \alpha$), is a measure of the range of singularity strengths in a multifractal system. It provides insight into the complexity and heterogeneity of the system. A larger width indicates a more complex and heterogeneous structure, while a smaller width suggests a more homogeneous structure. $f(\alpha )$ figure for all cases with different EM wave amplitudes have a concave diagram, which is in agreement with the multifractal nature of the system (Fig. 10 b). In Fig. 10 b, $\Delta \alpha$ have a greater value for high-amplitude irradiations, which confirms a more complex system than other cases.

**Fig. 11:**
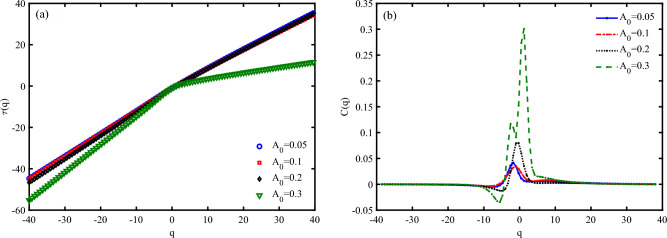
Multifractal analysis of electronic states a P53 sequence with $N=120~bps$ for different irradiation amplitudes in the unit of $\frac{\hbar c}{e r_{0}}$, (**a**) mass exponent function $(\tau (q))$, and (**b**) analogous specific heat (*C*(*q*)), ($\omega =0.1~THz$, $S_{l}=-4$).

Figure 11 a, represents mass exponent function ($\tau (q)$) for different values of $A_{0}$. Deviations from a straight line in $\tau (q)$ diagram indicate multifractality of system, where different parts of the time series scale differently and have multiple scaling exponents. The greater the deviation from the straight line, the higher the degree of multifractality. $\tau (q)$ diagram confirms that the system bring a high degree of multifractality through increase the irradiation amplitude (Fig. 11 a). Then, we find that diminishing the electrical current of a DNA piezoelectric system via the increase the amplitude of irradiation can be due to the enhancement of multifractality and then, complexity of the system.

In multifractal analysis, the concept of “analogous specific heat” ( *C*(*q*) ) is used to describe the variability of the multifractal spectrum^54^. It is analogous to the specific heat in thermodynamics, which measures the response of a system to changes in temperature. Here, *C*(*q*) measures the response of the multifractal spectrum to changes in the moment order *q*. *C*(*q*) equation represents the second derivative of the mass exponent function concerning *q*, analogous to the specific heat in thermodynamics. *C*(*q*) provides a measure of the variability or “heat capacity” of the multifractal spectrum. A higher *C*(*q*) indicates greater variability in the scaling behavior of the time series. Also, *C*(*q*) curve can resemble a classical phase transition, with peaks indicating critical points where the scaling behavior changes significantly. *C*(*q*) determines a classical (first-order) physical phase transition at a critical point indicated by the primary peak position at $A_{0}=0.05,0.1, 0.2~\frac{\hbar c}{e r_{0}}$. Additionally, *C*(*q*) exhibits a shoulder to the left of the primary peak at $A_{0}=0.3~\frac{\hbar c}{e r_{0}}$ (Fig. 11 b). It is known that such unusual behavior of a double-peaked specific heat function is observed in the Hubbard model within the weak-to-strong coupling regime. Consequently, it can be inferred that the presence of the principal peak in *C*(*q*) signifies a similar phase transition that may occur when the system responds to particular drives. The double peak behavior under nonspecific loading is associated with the onset of a crash in the electrical current as an output signal.

## Discussion

The piezo-vibro(photo)tronic effect in DNA is a fascinating and relatively unexplored area compared to its application in traditional piezoelectric materials like ZnO, GaN, and InN^55,56^. This work diverges from conventional piezo-phototronics by employing DNA’s unique mechano-optoelectronic properties in THz wave irradiation^57^. In conventional materials (e.g., ZnO, GaN), the piezo-phototronic effect arises due to the wurtzite crystal structure, which generates a piezopotential under strain. DNA, however, lacks a crystalline structure but exhibits charge transport properties influenced by mechanical strain and external irradiation. This study explores how these effects modify electrical current in DNA chains. In semiconductors, piezoelectric charges modulate carrier generation and recombination, enhancing optoelectronic device performance^58^. Traditional piezo-phototronic materials are used in LEDs, solar cells, and photodetectors. DNA-based piezo-vibro(photo)tronics could lead to biocompatible sensors, molecular electronics, and bio-optoelectronic devices, expanding the scope of piezo-phototronic applications^59,60^. Whereas prior studies required piezoelectric semiconductors (e.g., ZnO), we achieve strain-gated photoresponse in a biomolecular system, operating at THz frequencies to preserve DNA integrity. Unlike UV/visible-driven phototronics, we target THz frequencies ($0-2~$THz) to exploit DNA’s low-energy vibrational modes, avoiding bond-breaking damage. The current model predicts strain-sensitive electrical current modulation, while enabling biological compatibility unmet by inorganic platforms. The current work bridges the gap between biological charge transport and piezo-phototronic principles, offering a fresh perspective on molecular electronics. In our model, we simulate THz irradiation via a time-dependent vector potential, which couples to the electronic hopping term through a Peierls phase. This formulation enables coherent light–lattice coupling in the THz regime. Importantly, we do not explicitly include quantized phonon modes; rather, we treat vibrational modes semiclassically via the Peyrard–Bishop–Dauxois (PBD) model for base pair stretching and the tight-binding model for electron transport.

Although the DNA molecule indeed supports discrete low-energy vibrational modes (typically in the 0.1–1.5 THz range), several factors contribute to the smoothed or broadened response observed in the current–frequency plots included: resonance broadening, weak coupling regime, and superposition of multiple close-lying modes. Therefore, resonance broadening due to finite pulse width $\tau$, which introduces spectral broadening (Fourier-limited), damping and nonlinearities inherent in the DNA mechanical response (e.g., from the Morse potential and stacking interactions), which spread resonance peaks, and electron–lattice interaction terms, which modulate hopping via $e^{-p y_n}$, acting as a nonlinearity and suppressing sharp features. The amplitude of the driving field $A_0$ is intentionally kept in a moderate regime to avoid unphysical overheating or bond breaking. As a result, while resonant enhancement does occur, it is not sharply peaked, but instead manifests as enhanced oscillatory behavior over a range of nearby frequencies. On the other hand, DNA supports a dense set of collective modes, especially in long sequences like the 120-bp P53 strand studied here. The observed current response effectively averages over multiple overlapping vibrational modes, especially when the THz field simultaneously excites more than one.

While theoretical results can typically be validated against experimental findings, no direct experimental evidence exists for the piezo-phototronic effect in DNA. Therefore, we compare our results with experimental data from other semiconductor materials exhibiting similar effects. Additionally, we examined studies of photoexcitation in DNA to verify that our model’s trends remain consistent with established phenomena^61,62^.

To theoretically validate the reliability and applicability of our photo-induced charge transport model in DNA, we first verified that the tight-binding Hamiltonian correctly incorporates: (1) base-pair hopping terms under mechanical strain, (2) photon-matter interactions, (3) piezoelectric modulation of both on-site and hopping energies, and (4) gauge invariance in the Peierls substitution describing photon coupling. Subsequently, we compared the calculated electrical current with previous theoretical findings. Recent studies have demonstrated that the piezo-phototronic effect can significantly enhance the performance of wurtzite-structured semiconductors-such as ZnO, GaN, InN, and CdS-in photodetector applications by enabling strain-engineered control of charge carriers. These materials uniquely integrate three fundamental properties: piezoelectric response, semiconducting behavior, and photoexcitation capability. The piezo-phototronic mechanism functions through strain-induced piezoelectric polarization charges at interfaces and junctions, which dynamically alter the band structure and precisely regulate charge carrier dynamics-including generation, separation, transport, and recombination-across optoelectronic processes^11^. Strain-induced piezoelectric polarization fields modulate the Schottky barrier height at metal–semiconductor interfaces, enhancing responsivity by up to ten orders of magnitude^63^. Mechanical strain shifts the band-edge absorption through the deformation potential, enabling real-time adjustment of the spectral range^64^. Piezo-phototronic gating reduces carrier transit time in photodetectors, thereby enhancing device speed and overall performance^65^. The integration of piezoelectric and photovoltaic effects further enables battery-free photodetection with strain-tunable gain. Moreover, heterostructures exhibit strain-enhanced photoconductivity, making them particularly well-suited for biocompatible sensor applications^66^. The mechano-optoelectronic properties of junctions enable highly efficient molecular photoswitching, offering precise control over charge and energy flow at the molecular scale^67^. Therefore, the piezo-phototronic effect presents a promising strategy for enhancing the performance of photodetectors based on wurtzite-structured materials. It holds significant potential for applications in optical communication, advanced optoelectronic devices, and multifunctional computing systems. The performance metrics of DNA nanowire-based devices, such as photodetectors and solar cells, were significantly improved under the influence of the piezo-phototronic effect. The external quantum efficiency and responsivity of these devices showed marked enhancements with applied strain^68^. The results indicate that the piezo-vibro(photo)tronic effect can be effectively utilized to enhance the performance of DNA-based optoelectronic devices, making them viable for applications in flexible and wearable electronics^69^.

The ability to tune the electronic properties of DNA nanowires through mechanical strain opens new avenues for the design of strain-sensitive sensors and actuators.

Piezo-phototronic effect enhanced broadband photosensing using the the Piezoelectric polarization charges (piezocharges), arise due to mechanical strain. The essence of piezo-phototronics lies in the piezoelectric potential generated within piezoelectric semiconductor materials, which exhibit piezoelectricity, semiconductor properties, and photoexcitation characteristics^1,70^.

In the absence of external stress, the centers of cations and anions overlap, resulting in a macroscopically uncharged state. When stress is applied to the system, the charge centers of the cations and anions are displaced relative to each other, creating a dipole moment. The cumulative effect of the dipole moments generated by all the cells in the crystal produces a macroscopic potential distribution along the direction of the applied stress, known as the piezoelectric potential^6,71^. The piezo-phototronic effect play an important role in optical device performance^15,16^. Despite the increasing demand for photodetectors, achieving a broadband response with high sensitivity, rapid response times, and robust stability remains challenging. These qualities are essential for applications in optical communication, advanced multispectral detection, and environmental monitoring^18^. Piezo-phototronic properties of nanowires allows tuning and controlling of electro-optical process by strain-induced piezopotential. It also facilitates greater integration between piezoelectric devices and micro-electronic and optomechanical systems. It is fascinating to show how the piezo-phototronic effect can significantly enhance the responsivity of a photon detector across the entire spectrum from visible to UV. The piezo-phototronic effect can increase the detection sensitivity of light detection devices by more than five times. Adjusting the band structure, particularly at the p–n junction, through piezoelectric polarization charges is effective in improving the performance of optoelectronic devices^72^. Near-infrared (NIR) wavelength (THz) sensing has garnered significant attention due to its wide range of applications in biological imaging, communications, environmental monitoring, medical treatment, spectroscopy, security, and more. Over the past few decades, various NIR photodetectors with excellent photoresponse performance have been developed using single-crystalline InGaAs^73^, PbS quantum dots^74^, two-dimensional layered materials^75^, organic semiconductors^76^, photomultiplier tubes^77^, and others. However, many of these devices face challenges such as high cost, complex fabrication processes, and stringent operational requirements. Additionally, achieving an optimal balance between photoresponsivity and response speed remains a challenge for further expanding the practical applications of NIR PDs^78^. In this study, we investigate the effects of terahertz (THz) irradiation on DNA sequences to analyze their response to photoexcitation. The $0.02-2~$THz frequency range was selected to target the intrinsic mechanical resonances of DNA, where strain and electromagnetic (EM) fields act synergistically to enhance charge transport. At higher frequencies (e.g., $>10~$THz), electronic excitations dominate, thereby diminishing the effectiveness of piezo-phototronic gating. To address this, we employed the piezo-vibronic effect to achieve THz-driven strain coupling in DNA. Our results support the existence of this emerging phenomenon in DNA chains. The piezo-vibronic effect thus opens new avenues for innovative applications in next-generation optoelectronic technologies. Our model is theoretical in nature and focuses on idealized conditions: a single DNA chain (p53 sequence) connected between metal leads and subjected to externally applied mechanical strain and THz-frequency electromagnetic radiation ($<2~$THz). The purpose of referencing experimental literature is not to claim direct quantitative agreement, but rather to show that each key physical mechanism used in our model has been observed independently in prior experimental studies, even if under different configurations. To bolster the theoretical model, we now explicitly connect it to prior experimental studies on DNA optoelectronics and strain response. While direct experimental validation of THz-frequency piezo-phototronics in DNA is limited, our model aligns with: (i) strain-dependent conductance measurements in DNA and piezoelectric polarization^21,42,79,80^, validates that mechanical deformation modulates electrical response in DNA, (ii) THz-interaction with DNA^40,81^, demonstrates that THz photons can drive low-energy vibrational excitations, (iii) photocurrent enhancment in DNA hybrids^23^, shows that combined strain and light can modulate DNA conductivity in hybrid devices. We use transitionmetal dichalcogenides (TMDs) for explaining some of the observations, since TMDs^82^ and DNA both exhibit strain-tunable bandgaps (via deformation potential), and piezoelectricity in non-centrosymmetric structures. Also, TMD studies validate the tight-binding methods adapted here for DNA^33^. Therefore, comparisons to TMDs are justified by their shared mechano-optoelectronic mechanisms.

## Conclusion

The current study demonstrates the significant impact of the piezo-vibro(photo)tronics effect on the electronic properties of DNA chains. By applying mechanical strain, we observed notable changes in the charge transport structure of DNA, which were further modulated by photonic excitation. Electromagnetic (EM) irradiation enhances the electrical current through DNA, indicating that the electrical response of DNA to photon excitation manifests as an amplified current. Additionally, the application of mechanical strain further increases current amplitude, with values reaching up to 20 nA at specific frequencies. Under compressive strain, the electrical current is higher compared to tensile (stretching) strain. This enhancement persists as long as the tilt angle $(\alpha )$ remains below $\pi /4$ radians. For $\alpha > \pi /4$, the electrical current diminishes with increasing $\alpha$, eventually approaching zero.

Furthermore, the *I*–*V* characteristics of the P53 DNA sequence at various lengths confirm that electrical current is inversely proportional to chain length. Among the tested configurations, the DNA sequence with $N = 60~$base pairs exhibits the highest current peak. These findings highlight the potential of DNA as a versatile material for nanoscale optoelectronic devices, where strain-induced polarization and photonic interactions can be harnessed to enhance device performance. The insights gained from this research pave the way for future exploration of DNA-based materials in advanced technological applications, offering a new avenue for developing of innovative nanoscale electronic and optical systems.

## Data Availability

Data sets generated during the current study are available from the corresponding author on reasonable request.
